# The regulatory role of PGC1α‐related coactivator in response to drug‐induced liver injury

**DOI:** 10.1096/fba.2020-00003

**Published:** 2020-07-11

**Authors:** Marcin Buler, Thomas Naessens, Johan Mattsson, Yannick Morias, Magnus Söderberg, Philip Robbins, Lillevi Kärrberg, Tor S. Svensson, Petra Thulin, Björn Glinghammar, Richard C. Scarpulla, Ulf Andersson

**Affiliations:** ^1^ Clinical Pharmacology and Safety Sciences AstraZeneca R&D Mölndal Sweden; ^2^ Citizen Scientist Bruce ACT Australia; ^3^ Science for Life Laboratory Drug Discovery & Development Platform & Division of Translational Medicine & Chemical Biology Department of Medical Biochemistry and Biophysics Karolinska Institutet Stockholm Sweden; ^4^ Department of Cell and Molecular Biology Northwestern Medical School Chicago IL USA

**Keywords:** cytokine expression, drug‐induced liver injury, hepatic inflammation, PPRC1, PRC

## Abstract

PGC1α‐Related Coactivator (PRC) is a transcriptional coactivator promoting cytokine expression in vitro in response to mitochondrial injury and oxidative stress, however, its physiological role has remained elusive. Herein we investigate aspects of the immune response function of PRC, first in an in vivo thioacetamide (TAA)‐induced mouse model of drug‐induced liver injury (DILI), and subsequently in vitro in human monocytes, HepG2, and dendritic (DC) cells. TAA treatment resulted in the dose‐dependent induction of PRC mRNA and protein, both of which were shown to correlate with liver injury markers. Conversely, an adenovirus‐mediated knockdown of PRC attenuated this response, thereby reducing hepatic cytokine mRNA expression and monocyte infiltration. Subsequent in vitro studies with conditioned media from HepG2 cells overexpressing PRC, activated human monocytes and monocyte‐derived DC, demonstrated up to 20% elevated expression of CD86, CD40, and HLA‐DR. Similarly, siRNA‐mediated knockdown of PRC abolished this response in oligomycin stressed HepG2 cells. A putative mechanism was suggested by the co‐immunoprecipitation of Signal Transducer and Activator of Transcription 1 (STAT1) with PRC, and induction of a STAT‐dependent reporter. Furthermore, PRC co‐activated an NF‐κB‐dependent reporter, indicating interaction with known major inflammatory factors. In summary, our study indicates PRC as a novel factor modulating inflammation in DILI.

**Abbreviations**
ALT: alanine aminotransferase
AST: aspartate aminotransferase
DC: dendritic cells
DILI: drug‐induced liver injury
NF‐κB: nuclear factor kappa‐light‐chain‐enhancer of activated B cells
PRC: PGC1α‐related coactivator
STAT1: signal transducer and activator of transcription 1
TAA: thioacetamide
TASO: thioacetamide *S*‐oxide

## 1 INTRODUCTION

PGC1α‐Related Coactivator (PRC, PPRC1), together with PGC1α and PGC1β (Peroxisome proliferator‐activated receptor gamma coactivator 1‐alpha and beta), forms a small family of transcriptional coactivators which control mitochondrial biogenesis and energy expenditure.

In general, transcriptional coactivators regulate broad networks of genes^1^ by activating the gene expression through interactions with nuclear receptors.^2^ Indeed, PRC was originally identified as a transcriptional coactivator linking cellular proliferation to mitochondrial biogenesis.^3^ Later it was demonstrated that oxidative stress in U2OS cells induces PRC, which subsequently coactivates the expression of pro‐inflammatory cytokines.^4^, ^5^ However, PRC function in vivo has not been well described, as all mouse homozygous knockouts die during the preimplantation stage.^6^

Inflammation of the liver is a manifestation of a variety of diseases, including viral infection, nonalcoholic steatohepatitis, and drug‐induced liver injury (DILI). The pro‐inflammatory cascade that follows hepatocyte death, whereby immune cells react to the apoptotic bodies and to the cytokines released from moribund hepatocytes, is a hallmark of this condition. The degree of immune system activation, for example, the number and type of infiltrating cells, can influence the severity of the injury as monocytes and neutrophils are rapidly recruited to the injury site and could either exacerbate the ongoing tissue damage or alternatively drive the resolution of liver injury.^7^, ^8^

An interrupted immune response modifies DILI. For instance, mouse knockout of the Ccl2 gene, which is a potent monocyte chemoattractant, or of the Tnfα receptor (Tnfr1/55), protects mice from carbon tetrachloride‐(CCl_4_)induced liver injury.^9^, ^10^ In contrast, acetaminophen‐induced hepatotoxicity was exacerbated in Tnfr1/55 ‐/‐ mice.^11^ Such differences indicate that the beneficial or detrimental effect of immune system activation in DILI is multifactorial, such as the dose, genetic background of the in vivo model, and the mechanism of action of the toxicant.

**FIGURE 1:**
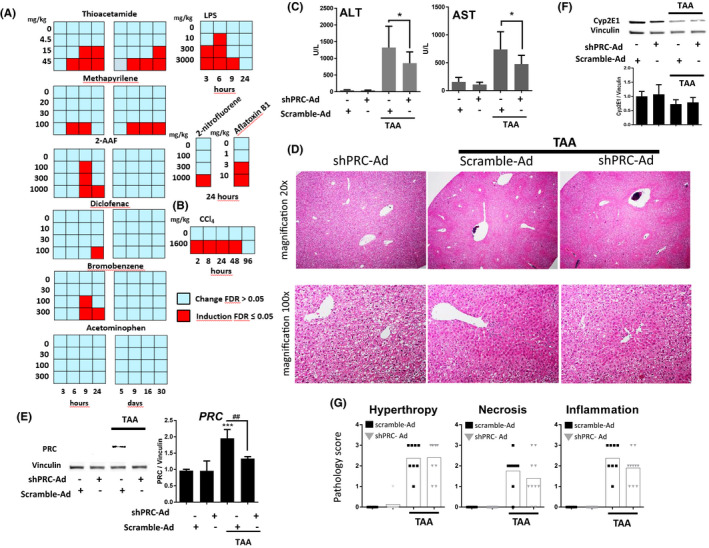
Prc mRNA expression profiles in rats and mice treated with hepatic toxicants and effect of adenovirus‐mediated Prc knockdown on liver injury in TAA‐treated mice. (A) rat and (B) mouse hepatic Prc mRNA microarray expression profiles in animals treated with hepatic toxicants as indicated. Each square represents a group treated with different dose (mg/kg) and sacrificed at a different time point. Only statistically significant induction is marked (red squares, FDR ≤ 0.05), see^20^ for experimental details. Fold changes are omitted. (C‐G) Male mice were infected with scramble or shPRC adenovirus (3 × 10^12^ VP/kg) for 48 h and treated with 100 mg/kg TAA for 24 h before sacrifice. (C) Plasma AST and ALT levels in animals from two independent experiments, one tailed t test, n = 7‐9, *P < .05, SD (D) Hematoxylin‐eosin stained representative sections of the liver from the animals treated as indicated. (E and F) Western blots of hepatic protein probed with indicated antibodies. Vinculin was used as a loading control and quantification of Vinculin normalized PRC and Cyp2E1 levels are shown. ANOVA, Tukey′s test, n = 3‐4, * denotes statistical significance to saline injected scramble‐Ad group while # relates to scramble‐Ad or shPRC‐Ad treated with TAA, ***P < .001, ##*P* < .01, SD (G) Histopathological evaluation of livers from two independent experiments, scoring was done as described in the Materials and methods section. Statistical significance was not calculated

Oxidative stress is a common feature both in disease and DILI. In fact, a disproportionate number of drugs that have been removed from the market due to DILI have been shown to interfere with some aspect of mitochondria,^12^ which is a primary intracellular source of reactive oxygen species (ROS). Similarly, oxidative stress is a characteristic response to several hepatic toxicants, including: Thioacetamide (TAA,14), Methapyrilene,^13^ Diclofenac,^14^ CCl_4_,^15^ Bromobenzene,^16^ 2‐Acetylaminofluorene (2‐AAF,^17^), Lipopolysacharide (LPS,^18^), and Aflatoxin B1.^19^ Indeed, interrogation of the public transcriptomics rat liver dataset from the Toxicogenomics Informatics Project (TG‐GATE, http://toxico.nibiohn.go.jp ^20^), revealed that PRC mRNA induction in vivo, especially within the first 24 hours, correlates with treatment with the aforementioned compounds (Figure 1A,B). Surprisingly, acetaminophen treatment in these rat studies did not result in any significant increase in PRC mRNA expression.

While it is clear that oxidative stress can activate NF‐κB signaling,^21^ the factors initiating the inflammatory cascade in hepatocytes remain poorly described. Considering the effect PRC has on cytokine expression in response to ROS and mitochondrial damage in U2OS cells, we hypothesized that inducible PRC may regulate hepatic inflammation in DILI.

This study provides the first evidence that PRC induced by hepatic toxicants and metabolic inhibitors can control cytokine expression and activation of the immune system, both in vivo and in vitro.

## 2 MATERIALS AND METHODS

### 2.1 Materials

Thioacetamide (TAA), Oligomycin, DMSO, and CCCP were purchased from Sigma‐Aldrich. Vinculin #V9264, STAT1 #SAB4300326, and CYP2E1 HPA009128 were obtained from Sigma‐Aldrich, while p100/p52 #06‐413 from Millipore (Billerica, MA, USA) and p105/p50 #D4P4D from Cell Signaling (Danvers, MA, USA). PRC antibody was described previously.^5^ Anti‐mouse: CD45 (30‐F11), CD11c (HL3), Ly6c (AL‐21), Ly6g (1A8), MHCII I‐A/I‐E (M5/114.15.2), CD19 (ID3), CD8 (53‐6.7), CD4 (RM4‐5), TCRβ (H57.597), CD11b (M1/70) were purchased from BD Biosciences and F4/80 (BM8) from eBioscience. Anit‐human: CD14‐v500, CD1a‐FITC, HLA‐DR‐APC‐H7, CD86‐BV421, CD83‐PE were acquired from BD Biosciences and CD40‐APC from eBiosciences.

### 2.2 TASO synthesis

Hydrogen peroxide (30% w/w, 580 µL, 6.32 mmol) was added dropwise to a solution of TAA (500 mg, 6.66 mmol) in MeOH (10 mL) at 0°C. The reaction was stirred at 0°C. NMR showed 89% conversion with respect to TAA after 1 h. Subsequently, after 2 hours, the solvent was evaporated (the water bath temperature was set to 30°C). The residue was purified by automated flash chromatography on a Biotage® KP‐SIL 25 g column. A gradient from 10% to 25% of MeOH in EtOAc over 12 CV was used as mobile phase. The product was collected using the wavelength 230 nm. The pooled fractions were evaporated (the water bath temperature was set to 30°C). The compound obtained, thioacetamide *S*‐oxide (521 mg, 86%), was found to be a white solid which was stored at –20°C. ^1^H NMR (500 MHz, DMSO) δ 1.95 (s, 3H), 8.14 (s, 1H), 8.96 (s, 1H). ^13^C NMR (126 MHz, DMSO) δ 13.8, 189.9.

### 2.3 Pathology

Liver samples were fixated in buffered 10% formaldehyde. After fixation, sections of the tissues were dissected out and blocked in paraffin. From the paraffin blocks 5 micron thick sections were made, mounted on glass slides and further deparaffinized and dehydrated according to standard protocols. Slides were stained with routine hematoxylin‐eosin and cover slipped before histological examination. Degrees of hepatocellular hypertrophy, hepatocellular necrosis and lobular inflammation were scored semi‐quantitatively on a five‐degree scale from 0 (none) to 4 (severe). Digital photomicrographs were obtained with an Olympus SC50 camera mounted on a Zeiss Axioskop 40 microscope.

### 2.4 Protein arrays and cell culture cytokine measurement

Cytokine levels in cell culture supernatants from HepG2 cells overexpressing PRC for 48 hours were measured with V‐PLEX Cytokine Panel (Rockville, MD, USA) or Human CCL5/RANTES Quantikine ELISA Kit (R&D Systems, Inc, Minneapolis, MN, USA) according to the manufacturer's protocol.

### 2.5 Differentiation of human monocyte‐derived DC

Peripheral blood mononuclear cells (PBMCs) from healthy donors were separated from peripheral blood by Ficoll‐Paque PLUS gradient (GE healthcare) centrifugation. CD14+ monocytes were subsequently isolated via negative selection using the Human Monocyte isolation kit II (Miltenyi Biotec) according to the manufacturer's protocol. For DC differentiation, purified monocytes were incubated in RPMI 1640 medium (Life Technologies) supplemented with 10% heat‐inactivated FBS, 2 mmol/L L‐glutamine, 1 mmol/L pyruvate, 100 U/mL penicillin/streptomycin (all from Thermo Fisher Scientific), 100 ng/mL human rGM‐CSF, and 40ng rIL‐d on day 7. Subsequently, cells were cultured in two time diluted media from HepG2 cells overexpressing PRC for 48 hours (RMPI 1640:DMEM 1:1; FCS 10%, P/S 1%). After 24 hours cells were collected an expression of selected surface markers measured as follow: Fc‐blocked cells (BD Biosciences) were stained with appropriate anti‐human antibodies in PBS (2% FCS, 5 mmol/L EDTA). 7‐AAD (BD Biosciences) was added to exclude death cells. Samples were acquired with FACS Canto and analyzed (FACS Diva software, BD Biosciences).

### 2.6 Plasmids and adenoviral vectors

Ha and Flag tags (N‐ and C‐terminal end, respectively), were added to PRC sequence by PCR reaction using FL‐PRC/pSV‐SPORT^3^ as a template and following primers: Fwd. 5′CTCGAGATGTACCCATACGATGTTCCAGATTACGCTGCGGCGCGCCGGGGACGG3′, Rev.5′GGATCCTTACTTATCGTCGTCATCCTTGTAATCCCTCCTGAGGTTCTTCT3′. PCR product was cloned into pCDNA3 vector between XhoI and BamH1 sites. PRC‐pcDNA3 was sequenced and no amino acid substitution, frameshift, or deletions were detected. Purified, in vivo grade, Scramble (Ad‐GFP‐U6‐scrmb‐shRNA (#1122N)) and shPRC (Ad‐GFP‐U6‐mPPRC1‐shRNA (shADV‐269267)) adenoviruses were obtained from Vector Biosystems Inc Reporter vector pGL4.32[luc2P/NF‐κB‐RE/Hygro] was purchased from Promega and expression levels measured with ONE‐Glo™ Luciferase Assay System (Promega). ISRE Cignal Reporter system was obtained from Qiagen and expression measured with Dual‐Glo® Luciferase Assay System (Promega).

### 2.7 Next generation sequencing and subsequent analysis

Quality of total isolated hepatic RNA was tested with Standard Sensitivity RNA Analysis Kit (Advanced Analytical Technologies) and library prepared with TruSeq® Stranded mRNA HT (Illumina). Libraries were validated with Standard Sensitivity NGS Fragment Analysis Kit 1‐ 6,000bp (Advanced Analytical Technologies), pooled in equimolar concentrations, diluted and denatured according to Illumina guidelines. Samples were sequenced with NextSeq 500/550 using High Output v2 kit (Illumina) to average 9 million reads. Base calling were done by bcl2fastq from Illumina. Reads were mapped to mouse genome version mm10 with HiSat2 v.2.0.1‐beta and counts generated using featurecounts (version 1.4.4) and TPMs were generated using Sailfish v. 0.7.6. Normalized counts were analyzed with Array Studio software (OmicSoft, Cary, NC, USA) and raw p‐values and False Discovery Rates (FDR) probability calculated. The set of genes with fold changes ≥ ±2 and p‐raw ≤0.05 were used for MetaCore pathway enrichment analysis.

### 2.8 Cell culture, compound treatment, and transfection

HepG2 (#HB‐8065) cells were obtained from ATCC and cultured in 1 g/L glucose DMEM, 5% Fetal Calf Serum (FCS), and % penicillin/streptomycin (P/S, Thermo‐Fisher Scientific). Twenty‐four hours after plating and counting with NC‐3000 (Chemometec), medium was replaced with DMEM (1 g/L glucose, no FCS, no P/S) Supplemented with compounds as indicated. After an additional 24 hours cells were collected. Alternatively, 24 hours after plating, cells were transfected with control endoribonuclease prepared siRNA (esiGFP or esiLUC) or esiPRC using Xtreme siRNA transfection reagent (Sigma‐Aldrich) according to the manufacturer′s protocol. After 24 hours oligomycin or CCCP were added and after additional 24 hours cells and culturing media were collected. For immunoprecipitation and reporter experiments cells were plated in DMEM medium (1 g/L glucose, 5%FCS and 1%P/S), transfected as indicated with Lipofectamine 3000 (Thermo‐Fisher Scientific) and collected 48 hours later. pcDNA3 or pGAL vectors were used either as controls or to normalize DNA load.

### 2.9 Animal handling

Ten‐week‐old female (TAA dose study) or 8‐week‐old male (adenovirus experiments) male C57BL/6N mice (Charles River, Germany), were fed with regular chow diet and had free access to tap water during all times. Temperature was maintained between 21°C and 22°C with relative humidity of 50 ± 5%, 20 air changes per hour and standard day/night cycle. Mice were housed in non‐sterile open cages (Makrolon III; Scanbur) containing hardwood bedding with nesting material (Papyrus, Mölndal, Sweden), gnawing sticks, and a shelter (Brogaarden). Female mice were group‐housed, while male mice were single‐housed. Animals were acclimatized for at least 5 days before the procedure. Mice were not fasted before a challenge and all procedures were performed during light cycle.

### 2.10 Animal studies

All experiments were conducted using protocols approved by the regional animal welfare committee (Göteborg Djurförsöksetiska Nämnd) in accordance with Swedish regulatory requirements. Mice were randomized the day before the experiment and placed in restrainers during tail vein injections with Viral Particles (VP). After 48 hours mice were intraperitoneally injected with 100 mg/kg TAA or saline and sacrificed 24 hours later. Alternatively, uninfected mice were intraperitoneally injected with different doses of TAA or saline and sacrificed 24 hours later. The animals were terminated by exsanguination through cardiac bleed under isoflurane (Attane vet 1000 mg/g, Apoteket Sweden) and oxygen anesthesia. Blood was collected in Li‐Heparin coated tubes, centrifuged and ALT and AST measured with ABX Pentra ALT/AST CP reagents (Horiba Abx Sas). Liver samples from caudate, right medial and left lateral lobe were snap frozen in liquid nitrogen. Liver samples were homogenated with a Liver Dissociation Kit and gentleMACS™ Dissociator (Miltenyi Biotec) according to the manufacturer′s protocol. Isolated cells were resuspended in PBS (2% FBS/5 mmol/L EDTA), incubated with the FcR blocking antibody (2.4G2) for 5 minutes, followed by the Live/Dead Fixable Aqua Dead Cell Stain Kit (Thermo‐Fisher Scientific). Cells were stained with appropriate antibodies (Online materials and methods), acquired with BD LSRFortessa flow cytometer and analyzed with FlowJo (Tree Star) software. For histopathology studies, liver samples were fixated in buffered 10% formaldehyde. After fixation, sections of the tissues were dissected out and blocked in paraffin. From the paraffin blocks 5 μm thick sections were made, mounted on glass slides and further deparaffinized and dehydrated according to standard protocols. Slides were stained with routine hematoxylin‐eosin and cover slipped before histological examination. Degrees of hepatocellular hypertrophy, hepatocellular necrosis, and lobular inflammation were scored semi‐quantitatively on a five‐degree scale from 0 (none) to 4 (severe). Digital photomicrographs were obtained with an Olympus SC50 (Tokyo, Japan) camera mounted on a Zeiss Axioskop 40 (Darmstadt, Germany) microscope.

### 2.11 Protein isolation and western blots

Cells were lysed with RIPA or IP buffer (Sigma‐Aldrich). Liver samples were homogenized in RIPA with 5mm Stainless steel beads and TissueLyze homogenizer (Qiagen). Nuclear and cytosolic fractions were isolated with CellLytic Nuclear Extraction kit (Sigma‐Aldrich). Immunoprecipitation was performed with ANTI‐FLAG M2 Affinity Gel (Sigma‐Aldrich) according to the manufacturer's protocol. Alternatively, Protein G immunoprecipitation kit (Sigma‐Aldrich) and appropriate antibodies (p100/p52 or p105/p50) were used. All buffers were supplemented with Complete ULTRA proteasome inhibitor (Sigma‐Aldrich). Protein concentration was measured with Pierce BCA Protein Assay Kit (Thermo‐Fisher Scientific). After isolation, proteins were separated on 4%‐15% SDS‐PAGE gel (Bio‐Rad). Native PRC was transferred onto 0.2 µmol/L nitrocellulose membranes (Bio‐Rad) using Hoefer chamber (Holliston, MA, USA, 400 mA (constant), 73 minutes, Tris Glycine, 0.2% SDS). TransBlot Turbo system (Bio‐Rad) was used in experiments with recombinant PRC. Membranes were incubated in TBS Odyssey Blocking Buffer (Li‐Cor) and probed with appropriate antibodies diluted in blocking buffer Supplemented with 0.01% Tween20 (Sigma‐Aldrich). Afterward blots were incubated with anti‐rabbit IRDye 800CW and anti‐mouse IRDye 680RD 600 antibodies (Li‐Cor) diluted 1:20 000 (0.02% Tween20). Blots were developed using Odyssey CLx scanner and analyzed in Image Studio software (Li‐Cor).

## 3 RESULTS

### 3.1 PRC knockdown protects from TAA‐induced liver damage in mice

TAA is a hepatotoxic compound, used as a molecular tool to induce liver damage and fibrosis. As PRC mRNA expression was robustly activated by TAA in rat liver (Figure 1A, File S1, TG‐GATE, http://toxico.nibiohn.go.jp ^20^;), we explored in detail a mouse model of acute hepatic damage in the context of PRC induction. As expected, TAA‐induced Alanine Aminotransferase (ALT) and Aspartate Aminotransferase (AST) levels, hepatic cytokine expression, and was associated with monocyte and neutrophil infiltration in a dose‐dependent manner. These markers of ongoing liver inflammation and damage correlated with increased mRNA and protein levels of PRC (Figure S1A‐E).

Hepatocytes are the main target of adenoviral transduction during systemic delivery^22^ and are a major source of hepatic cytokines.^23^, ^24^ To investigate the role of PRC induction in response to a TAA insult, we reduced the hepatic PRC mRNA levels with an adenoviral vector carrying a specific shRNA sequence. In order to distinguish the effects of PRC knockdown from general adenoviral infection, we studied the effect of the adenovirus alone,^25^ and as expected, elevated expression of *Tnfα* and *Ccl5* were observed in infected mice, indicating ongoing inflammation independent of shRNA insertion (Figure S1F). However, in contrast to animals with a higher viral load (6 × 10^12^ VP/kg), treatment with 3 × 10^12^ VP/kg had no discernible effect on ALT and AST levels, suggesting negligible liver injury at this viral load (Figure S1G).

Subsequently, we investigated the effect of PRC knockdown in TAA‐induced liver damage. As indicated by elevated levels of the liver injury biomarkers ALT and AST, TAA (100 mg/kg) was indeed hepatotoxic (Figure 1C), with histopathological evaluation establishing that injury was limited to Zone 1 and located around central veins (Figure 1D). The shPRC‐Ad vector did not significantly affect constitutive expression of PRC protein, but clearly attenuated TAA‐mediated induction (Figure 1E). PRC knockdown modestly reduced the pathology scores for hepatic necrosis and inflammation, although there was no apparent effect on the hypertrophic response (Figure 1G). In keeping with the histopathological evaluation, ALT and AST levels were lower in animals treated with shPRC‐Ad and TAA (Figure 1C).

TAA is bioactivated and metabolized to thioacetamide‐S‐oxide (TASO) by Cyp2E1.^26^ Consistent with previous observations (Figure S1A), Cyp2E1 expression was reduced by TAA, but not affected by shPRC‐Ad (Figure 1F), indicating that the observed changes were not related to transcriptional downregulation of Cyp2E1 by PRC and subsequent inhibition of TASO formation.^21^

In summary, this set of data indicates that infection with shPRC‐Ad attenuates hepatic inflammation and necrosis and indicates that PRC contributes to TAA‐induced liver injury.

### 3.2 PRC knockdown reduces hepatic cytokine expression and leukocyte infiltration

**FIGURE 2:**
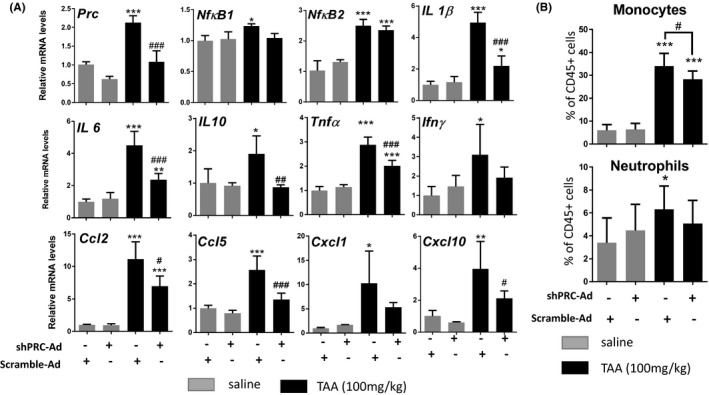
Adenovirus‐mediated Prc knockdown attenuates hepatic inflammation in TAA‐treated mice. (A) Animals were treated as described in Figure 3 and relative mRNA levels of selected genes were measured with qPCR. B) Relative number of monocytes and neutrophils in the liver presented as percentage of CD45+ cells. Combined results from two independent experiments are shown. ANOVA, Tukey′s test, (A) n = 3‐4, B) n = 7‐9 * denotes statistical significance to saline injected scramble‐Ad group while # relates to scramble‐Ad or shPRC‐Ad treated with TAA, *P < .05, **P < .01, ***P < .001, #*P* < .05, ## *P* < .01, ###*P* < .001, SD

To specifically study the effect of PRC knockdown on the immune system we measured hepatic cytokine and chemokine expression. The shPRC‐Ad infected animals exhibited a reduced expression of several hepatic cytokines and chemokines in response to the compound, for example, *Il1β*, *Tnfα*, *Ccl2*, *Cxcl1,* and *Cxcl10* mRNA levels were reduced by 55%, 31%, 37%, 48%, and 46%, respectively (Figure 2A). Neither *Nf‐κB1* or *Nf‐κB2* expression (Figure 2A), or p50 and p52 protein levels (unpublished data) were affected by PRC knockdown.

Furthermore, we investigated the functional aspect of cytokine and chemokine regulation by measuring the number of monocytes and neutrophils in the liver: In agreement with the mRNA expression profile, the shPRC‐Ad reduced both monocyte and (to a lesser degree) neutrophil infiltration in TAA‐injured liver (Figure 2B, Gating profile Figure S2A). However, numbers of eosinophils, dendritic cells and CD4+ T lymphocytes were not affected (data not shown). Similarly, expression of activation markers on monocytes (CD86, PD‐L1, CD40, and CD80) were not altered by shPRC‐Ad (Figure S2B).

Together, these results indicate PRC involvement in transcriptional regulation of inflammatory gene expression in mouse liver and suggest that PRC modulates cytokine expression during TAA‐mediated liver injury that potentially could affect the monocyte and neutrophil infiltration.

### 3.3 PRC controls HepG2 cytokine secretion and mediates human DC and monocyte activation

**FIGURE 3:**
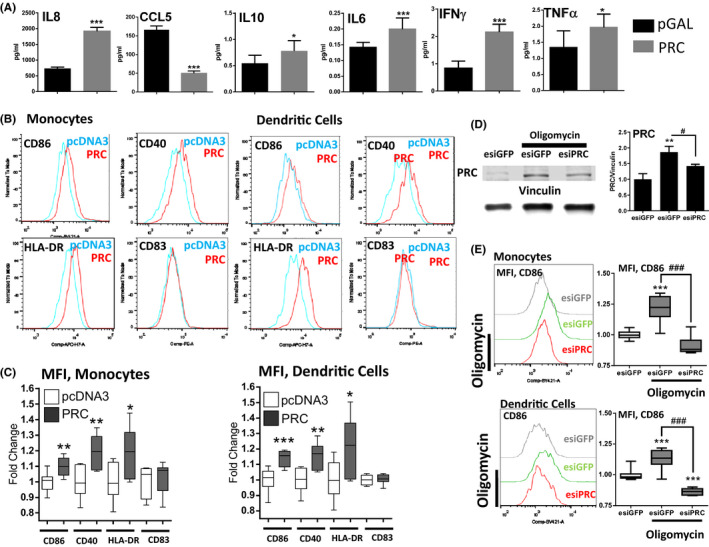
PRC controls cytokine expression and dendritic cell (DC) and monocyte activation by hepatocytes. (A) Levels of selected cytokines in culture supernatant were analyzed using MSD platform (V‐Plex) or ELISA (CCL5) in culturing media of HepG2 cells transfected with pGAL or PRC for 48 h. * denotes statistical significance to pGAL, ANOVA Dunnett's multiple comparisons test, n = 5‐8 *P < .05, **P < .01, ***P < .001, SD (B) Human monocytes and human monocyte derived dendritic cell were cultured for 24 h in supernatants from HepG2 cells overexpressing PRC for 48 h. Expression of activation markers: CD86, CD40, HLA‐DR, and CD83 was measured with flow cytometry (C) Quantification of Mean Fluorescence Intensity (MFI) from panel B. Statistical significance to pcDNA3 control samples is shown, Two tailed t‐test, *P < .05, **P < .01, ***P < .001, n = 7‐8. For this and all following box and whiskers graphs: The box always extends from the 25th to 75th percentiles, the whiskers go down to the smallest value and up to the largest, the line in the middle of the box is plotted at the median. Representative of two independent experiments with cells derived from two separate donors is shown. (D) PRC levels in HepG2 cells transfected with esiGFP or esiPRC for 24 h and treated with 0.04 µmol/L oligomycin for additional 24 h. Normalized quantification of PRC protein is shown E) Human monocyte derived dendritic cells and monocytes were cultured for 24 h in supernatants from HepG2 cells transfected with esiGFP or esiPRC1 for 24 hours and treated for additional 24 h with 0.04 µmol/L oligomycin. MFI is shown. Panels D and E: * denotes statistical significance to esiGFP control samples. # represents statistical significance between esiGFP and esiPRC samples treated with oligomycin. ANOVA, Tukey's test, F (n = 3) Representative of two independent experiments; panel E (n = 7‐8). Representative results of two independent experiments with cells derived from two separate donors is shown

In order to study the potential role of PRC in the regulation of cytokine expression in human hepatocytes, HepG2 cells were transiently transfected with a PRC expression vector for 48 hours and the cell culture supernatant analyzed for a panel of cytokines. As demonstrated in Figure 3A, PRC overexpression induced IL8, IFNγ, TNFα, IL6, and IL10 in cell culture supernatants, while robustly reducing CCL5 levels (3.3‐fold change). These data indicate that increased PRC expression is sufficient to modulate the expression of pro‐ and anti‐inflammatory cytokines also in the absence of toxicants.

To further demonstrate the physiological consequences of PRC‐dependent changes in cytokine secretion in hepatic cells, we isolated monocytes from the blood of healthy donors that were differentiated into DC for 7 days (Figure S3F). Subsequently, a mixture of undifferentiated monocytes (CD14^+^CD1a^‐^) as well as DC (CD14^+^CD1a^+^) were cultured in medium collected from HepG2 cells that had 48 hours prior been transfected with either a control vector, or PRC expression vector. Both monocytes and DC (Figure 3B,C) treated with culture supernatants from cells overexpressing PRC displayed higher expression of the surface activation markers CD86, CD40 and HLA‐DR, but not CD83.

Next, we investigated PRC response to TAA and its active metabolite––TASO––as well as oligomycin and carbonyl cyanide m‐chlorophenyl hydrazine (CCCP). While TAA proved ineffective, and its active metabolite TASO demonstrated only limited activation of PRC mRNA and protein in HepG2 cells, robust PRC induction was seen with oligomycin and CCCP, (Figure S4A‐E) as demonstrated previously in U2OS cells.^5^ This observation prompted us to use specific mitochondrial inhibitors as PRC inducers in order to investigate the direct effect of PRC in hepatocytes.

Subsequently, we investigated the effect of PRC knockdown in HepG2 cells cultured either with oligomycin (Figure 3D) or CCCP (Figure S3G). PRC esiRNA effectively blunted the oligomycin and CCCP‐mediated induction of PRC (Figure 3D and Figure S3G, respectively). Conditioned medium collected from oligomycin‐treated HepG2 cells‐induced CD86 expression on both DC and monocytes (Figure 3D), while medium of CCCP‐treated HepG2 cells only affected monocytes (Figure S3H). Importantly, this response was attenuated in both monocytes and DC when the PRC induction had been blunted by PRC esiRNA. CD83 was reduced by oligomycin and even further attenuated by esiPRC but remained unchanged in CCCP samples (Figure S3I,J). CD40 and HLA‐DR were not affected, while all markers were robustly upregulated in LPS treated samples (unpublished data). Taken together, these data indicate that induction of PRC expression is necessary for a functional pro‐inflammatory program also in human hepatoma cells that can translate into the activation of monocytes and DC.

### 3.4 PRC modulates NF‐κB and STAT1 signaling

**FIGURE 4:**
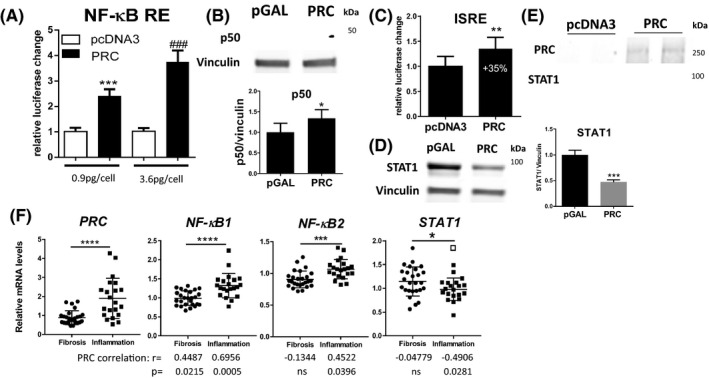
PRC modulates NF‐κB and STAT signaling. (A) HepG2 cells were co‐transfected with a luciferase reporter gene under the control of NF‐κB responsive elements and either pcDNA3 or PRC. Luciferase intensity was measured 48 h later. Representative of three independent experiments. ANOVA, Tukey's test, n = 11, ***P < .001, ###*P* < .001, SD. Symbols denote statistical significance to respective pcDNA3 control (* 0.9 pg/cell, # 3.6 pg/cell). (B) HepG2 cells were transfected with either pGAL or PRC vectors for 48 h, total cell lysates separated on SDS‐gel and expression of selected proteins assessed with indicated antibodies. (C) HepG2 cells were co‐transfected with ISRE reporter vector together with pcDNA3 or PRC and luciferase expression evaluated 48 h later. Two tailed t‐test. (D) STAT1 levels in HepG2 cells overexpressing PRC for 48 h. (E) PRC was immunoprecipitated from HepG2 cells overexpressing PRC for 48 h and probed with indicated antibodies. (F) RNA expression profiles from GDS4271 data set, Id_ref: *Prc* 203737_s_at, *NF‐κB1* 209239_at, *NF‐κB2* 209636_at, Stat1 200887_s_at. Two tailed t test, ****P < .0001, ***P < .001, *P < .05.Hollow square indicates an outlier (ROUT, Q = 5%). Values below graphs represent Pearson's correlation measures between Prc expression and selected gene in either fibrosis or inflammation category with corresponding statistical significance (two‐tailed)

Several pro‐inflammatory pathways converge in NF‐kB signaling, prompting us to test whether it is co‐activated by PRC. A PRC expression vector was co‐transfected with a luciferase reporter under control of tandem NF‐κB elements. PRC‐induced reporter gene expression in a dose‐dependent manner consistent with transcriptional co‐activation (Figure 4A). The NF‐κB pathway includes two routes: canonical, mediated by p105/p50 (NF‐κB1), and; noncanonical, mediated by p100/p52 (NF‐κB2). Both p105/p50 and p100/p52 must be cleaved to p50 and p52 and translocated to the nucleus to become transcriptionally active. We did not observe increased nuclear translocation of p50 or p52 after PRC overexpression, despite modestly elevated levels of p50 (Figure 4B). Likewise, we did not detect p50 or p52 in PRC immunoprecipitates (unpublished data). While a direct interaction between PRC and elements of NF‐κB pathway could not be demonstrated, the data suggests transcriptional co‐activation of NF‐κB.

Owing to PRC’s role as a transcriptional coactivator, we expected it to modulate a relatively large set of genes, as such, we performed RNA sequencing (RNAseq) of the mouse hepatic RNA isolated from the previous in vivo experiments to identify possible PRC‐dependent pathways. As expected, expression of several NRF2 target genes (*Nqo1*, *Srx1*, *Txnrd1)* were attenuated by shPRC‐Ad (Figure S4A,B), in agreement with the proposed PRC function as a regulator of oxidative stress response. Interestingly, expression of *Stat1* and *Stat2* transcriptional factors were downregulated by shPRC‐Ad in the RNAseq dataset, a result confirmed by qPCR analysis of the same samples (Figure S4C). Furthermore, several STAT downstream targets for example, *Mx2*, *Oas1a*, *Isq15*, *Ifit2* (FDR ≤ 0.05), were downregulated by shPRC‐Ad (File S4). Also, expression of Interferon regulatory factor 9 (*Irf9*), which together with Stat1 and Stat2 forms an Interferon‐stimulated gene factor 3 (*Isgf*3) complex, was reduced. In addition, Interferon regulatory factor 7 (*Irf7*) and several Interferon Sensitive Genes (ISG), for example, Interferon‐induced protein with tetratricopeptide repeats 1 and 44 (*Ifit1* and *Ifit44*), were attenuated by PRC knockdown with robust statistical significance (File S4), indicating that the coactivator is involved in STAT signaling.

Next, we investigated the PRC‐STAT1 interaction in more detail in human cells. Initially, we co‐transfected HepG2 cells with PRC and a reporter vector under the control of Interferon‐Stimulated Response Element (ISRE). We observed that PRC induced an ISRE‐mediated response (35%, Figure 4C), despite, paradoxically, simultaneously reducing STAT1 levels by 50% (Figure 4D). Interestingly, PRC co‐immunoprecipitated with STAT1 (Figure 4E), strongly suggesting a direct interaction between both molecules, even during conditions when STAT signaling is not activated.

As a final point, we searched publicly available GEO datasets for human hepatic diseases characterized by significantly increased Prc levels. Data from infants with biliary atresia characterized by an inflammatory signature, revealed 2.13‐fold higher Prc mRNA expression levels when compared to patients with a fibrotic profile (GDS4271^27^). Moreover, Prc levels correlated positively with elevated NF‐κB1 mRNA (r = 0.6956) in patients with the inflammatory molecular profile, and to lesser extent with those in the fibrotic category (r = 0.4487, Figure 4F). NF‐κB2 levels followed a similar pattern in infants with liver inflammation (r = 0.4522), while Stat1 expression was reduced and correlated negatively with Prc expression (r = −0.4906).

## 4 DISCUSSION

In this study we have shown that PRC is involved in stimulating a pro‐inflammatory response in hepatic cells during DILI. Our data indicates that PRC regulates the in vivo expression of several key pro‐inflammatory cytokines, including *Tnfα*, *Il‐1β,* and *Il16*, as well as the chemokines *Ccl2, Ccl5, Cxcl1,* and *Cxcl10*, responsible for attracting or activating neutrophils and monocytes.^7^, ^8^ Consistent with these observations, adenovirus‐mediated PRC knockdown attenuated monocyte infiltration and, to a lesser extent, neutrophil recruitment to TAA‐damaged liver. Moreover ALT, AST, and histopathological changes were reduced by shPRC‐Ad, indicating that the cytokine signaling it inhibits ameliorates TAA‐induced necrosis. Together, these results suggest that PRC regulates a toxicant‐induced, pro‐inflammatory program that aggravates the immune response and might contribute to corresponding hepatic response.

Mouse models do not replicate the human immune system in all aspects, for example, mice do not express IL8, which is instead functionally replaced by Cxcl1, Cxcl2, and Cxcl5. In addition, a response to TAA in vivo is pleiotropic and is not only limited to PRC‐mediated responses. Consequently, to establish whether the gene expression response to PRC induction was associated with a functional pro‐inflammatory response in isolation from toxicants, and in a human context, we performed an overexpression experiment in HepG2 cells. Human IL8 is a strong neutrophil attractant^28^ that together with IL6, IL10, IFNγ, and TNFα, was shown to be robustly induced by PRC‐overexpression in HepG2 cells. In contrast, CCL5 was strongly reduced in human hepatoma cells, with the opposite response in TAA‐treated mice. The variety of cytokines regulated by PRC indicate that they share a common, upstream transcriptional factor. Our results indicate that PRC activates the NF‐κB pathway in human HepG2 cells, while the cytokine response to TAA suggests a similar mechanism in the mouse model. This overall finding is in contrast with another study describing inhibition of NF‐κB signaling by PRC in human umbilical vein endothelial cells.^29^ However, we were unable to demonstrate a direct interaction between PRC and p50 or p52 (transcriptionally functional elements of the NF‐κB system), nor demonstrate their enhanced nuclear translocation after PRC overexpression, despite elevated levels of p50. As murine p50 or p52 levels were not affected by PRC knockdown in vivo, a more complex mechanism is likely involved that warrants further investigation.

Interestingly, shPRC‐Ad attenuated the expression of *Stat1* and *Stat2* in mouse liver, both of which regulate expression of several chemokines. For example, STAT1 drives the transcription of CXCL10 and CCL2,^30^ and regulates CCL5.^31^ We suspect that this reduced expression of STAT downstream targets is beneficial in mitigating TAA‐induced liver injury. Our in vitro results indicate that PRC interacts directly with STAT1 in HepG2 cells and can promote the transcription of genes under control of the ISRE element. STAT1, together with STAT2 and IRF9, forms an ISGF3 complex which is then only transcriptionally active in the context of ISRE.^32^ Incomplete activation or missing components of the complex during sterile (ie, not virally infected) transfection in HepG2 might explain the modest reporter induction observed, however, it does demonstrate that PRC is capable of activating ISRE genes independent of interferon signaling or viral infection. Interestingly, PRC overexpression reduced STAT1 levels, indicating a negative feedback loop. Clearly, the role of PRC in STAT signaling, especially during viral infection deserves an independent study. Nevertheless, our data, together with NF‐κB results, strongly suggests that PRC is involved in the regulation of at least two major immune signaling pathways. This notion was further strengthened by the fact that Stat1, NF‐κB1, and NF‐κB2 expression correlated with PRC levels in infants with inflammatory signature of biliary atresia.

Although our results from HepG2 cells and in vivo experiments indicate that PRC promotes inflammation, we could not reject a hypothesis that reduced levels of CCL5 or other, as yet unidentified, factors may counteract or even reverse this effect. Therefore, we evaluated the net effect of hepatic cytokines regulated by PRC on the effector immune system cells. Both monocytes and DC displayed increased expression of surface activation markers: CD86, CD40, and HLA‐DR, indicating that the response mediated by PRC is functional beyond hepatocytes and might activate lymphoid cells. Loss‐of‐function experiments combined with oligomycin and CCCP treatment further support this theory. Overall, our results indicate that the inflammatory program regulated by PRC in mice has a potential equivalent in humans.

The exact mechanisms involved in PRC induction are unknown^33^ and identifying factors regulating PRC expression, including specific nuclear receptors, were not in the scope of this study. It is also clear that not all hepatoxicants induce PRC, and that the toxicological mechanism of action likely contributes to the changes in PRC expression in response to different toxicants. In addition, as the action of the immune system differs depending on the model and the drug, as highlighted by the opposing liver toxicity outcomes of CCl4 and acetaminophen treatment in Trnf1/55 KO mice, the role of PRC is likely to be different depending on the context. Nonetheless, mitochondrial oxidative stress, induced by for example, CCCP, has previously been shown to increase PRC levels in vitro.^3^, ^4^, ^5^ This data has now been replicated in HepG2 cells and supported by further results with oligomycin, suggesting a general mechanism. TAA also directly inhibits the mitochondrial respiratory chain,^34^, ^35^ and, given that all compounds that have been identified to induce PRC mRNA expression, also compromise mitochondrial function, suggests that in vivo activation of the pro‐inflammatory program following PRC induction relates to oxidative stress. However, the direct mechanism regulating PRC levels remains to be investigated.

In conclusion, in this work we investigated the functional aspects of PRC and demonstrated that PRC mediates inflammation during DILI in mice, and in human hepatoma cells treated with mitochondrial toxicants. The activation of human DC and monocytes indicated that the inflammatory cascade initiated by PRC is functional in hepatocytes and beyond, and could contribute to various liver pathologies.

## AUTHOR CONTRIBUTIONS

M. Buler, U. Andersson designed research; M. Buler, U. Andersson, T. Naessens, M. Söderberg, Y. Morias analyzed data; M. Buler, T. Naessens, J. Mattsson, Y. Morias, L. Kärrberg, P.Thulin performed research, TS Svensson, R. C. Scarpulla contributed new reagents or analytic tools, M. Buler, U. Andersson, P. Robbins, B. Glinghammar wrote the paper.

## Supporting information

Fig S1

Fig S2

Fig S3

Fig S4

Table S1

File S1

File S2

File S3

File S4

Supplementary Material
