# Supplementary material for: The regulatory role of PGC1α‐related coactivator in response to drug‐induced liver injury
Source: FASEB Bioadv. 2020 Jul 11;2(8):453–63. doi: 10.1096/fba.2020-00003 (PMC7429352; doi:10.1096/fba.2020-00003)
Supplement: Supplementary file 1 — Fig S1 [file FBA2-2-453-s001.pptx]

## Slide 1
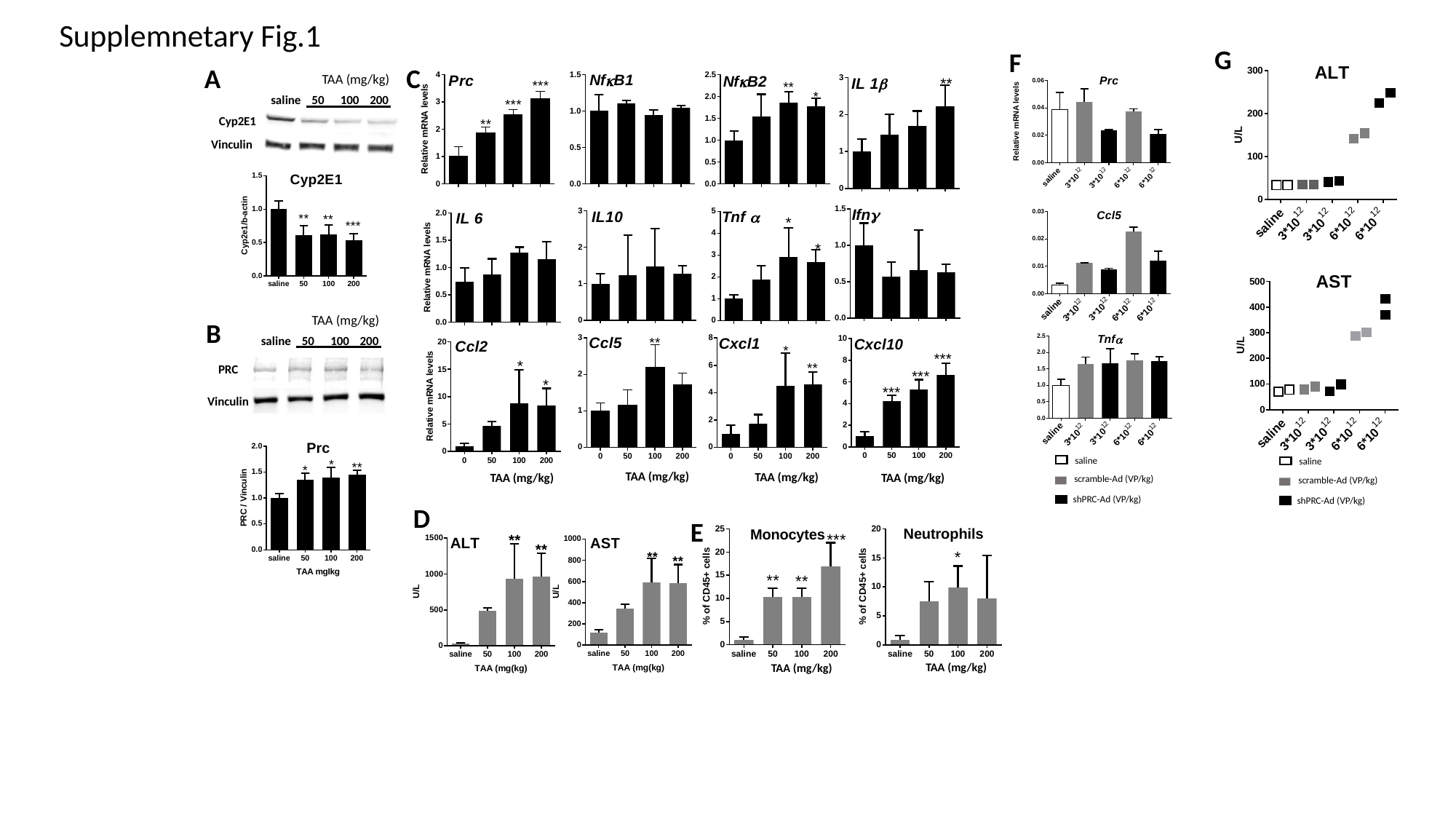

Supplemnetary Fig.1
G
F
A
C
TAA (mg/kg)
 saline 50 100 200
Cyp2E1
Vinculin
TAA (mg/kg)
B
 saline 50 100 200
PRC
Vinculin
saline
saline
TAA (mg/kg)
TAA (mg/kg)
TAA (mg/kg)
TAA (mg/kg)
scramble-Ad (VP/kg)
scramble-Ad (VP/kg)
shPRC-Ad (VP/kg)
shPRC-Ad (VP/kg)
D
E
TAA (mg/kg)
TAA (mg/kg)
