# Supplementary figures and images for: The regulatory role of PGC1α‐related coactivator in response to drug‐induced liver injury
Source: FASEB Bioadv. 2020 Jul 11;2(8):453–63. doi: 10.1096/fba.2020-00003 (PMC7429352; doi:10.1096/fba.2020-00003)

## Slide 1
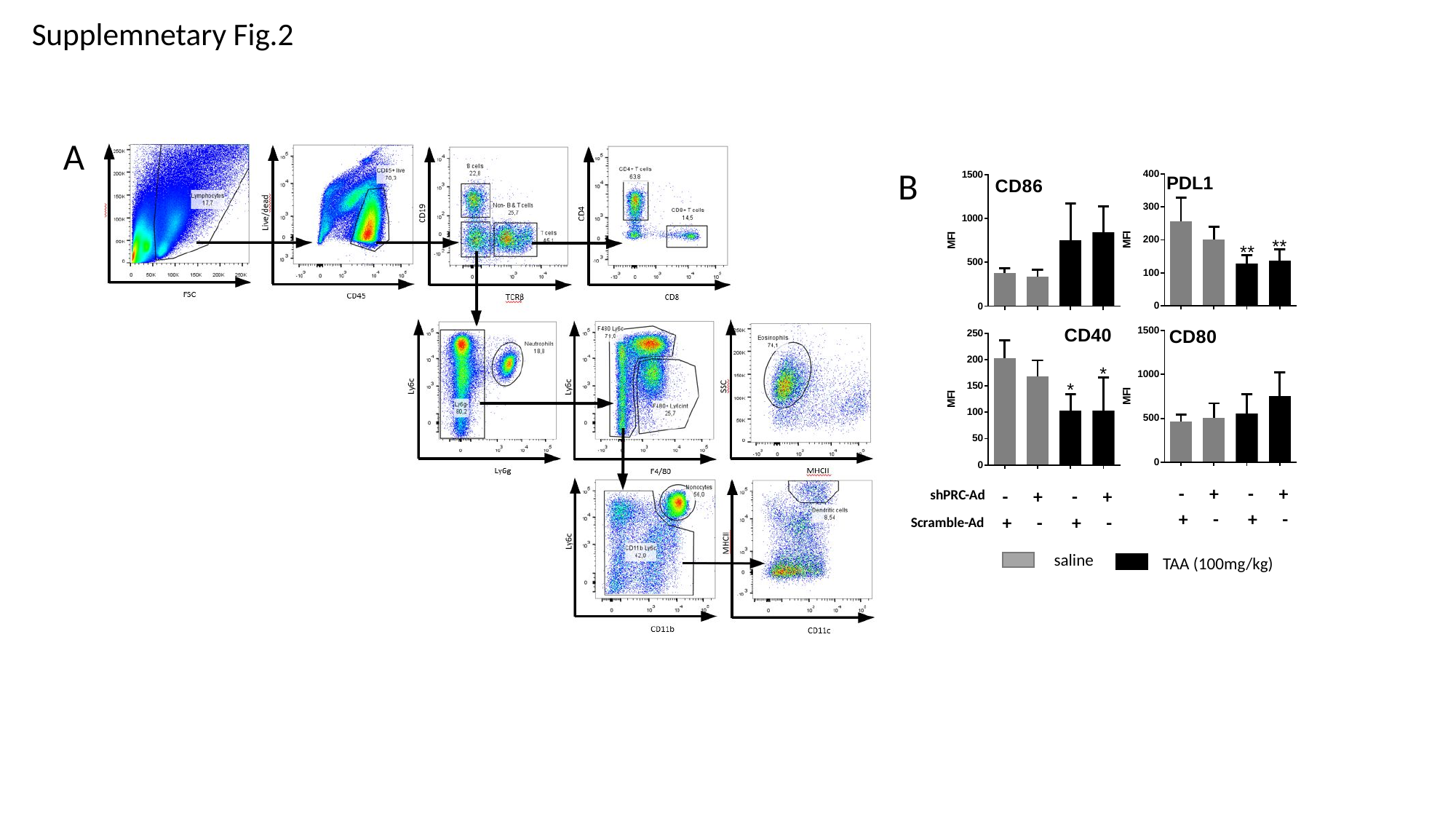

Supplemnetary Fig.2
A
B
 - + - +
 - + - +
shPRC-Ad
 + - + -
 + - + -
Scramble-Ad
saline
TAA (100mg/kg)

Supplement: Supplementary file 2 — Fig S2 [file FBA2-2-453-s002.pptx]
