# Supplementary material for: The regulatory role of PGC1α‐related coactivator in response to drug‐induced liver injury
Source: FASEB Bioadv. 2020 Jul 11;2(8):453–63. doi: 10.1096/fba.2020-00003 (PMC7429352; doi:10.1096/fba.2020-00003)
Supplement: Supplementary file 3 — Fig S3 [file FBA2-2-453-s003.pptx]

## Slide 1
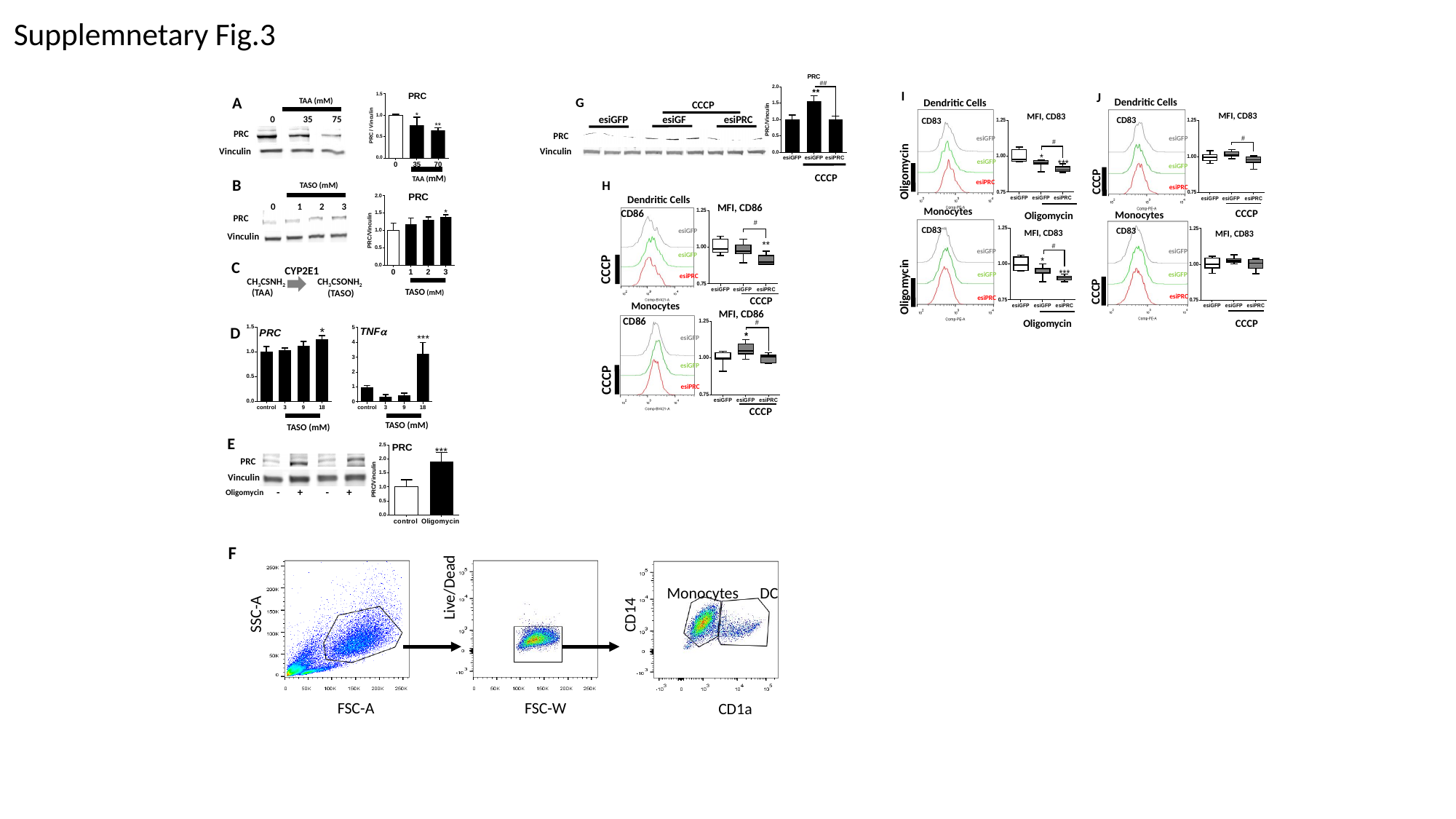

Supplemnetary Fig.3
I
J
Dendritic Cells
Dendritic Cells
MFI, CD83
MFI, CD83
CD83
esiGFP
esiGFP
esiGFP
esiGFP
Oligomycin
CCCP
esiPRC
esiPRC
Monocytes
Monocytes
CCCP
Oligomycin
CD83
CD83
MFI, CD83
MFI, CD83
esiGFP
esiGFP
esiGFP
esiGFP
Oligomycin
CCCP
esiPRC
esiPRC
CCCP
Oligomycin
TAA (mM)
A
G
TAA (mM)
CCCP
esiGFP
esiPRC
esiGFP
0 35 75
CD83
PRC
PRC
Vinculin
Vinculin
CCCP
B
H
TASO (mM)
Dendritic Cells
MFI, CD86
CCCP
CD86
esiGFP
esiGFP
CCCP
esiPRC
Monocytes
MFI, CD86
CD86
esiGFP
esiGFP
CCCP
esiPRC
CCCP
0 1 2 3
PRC
Vinculin
C
CYP2E1
CH3CSNH2 CH3CSONH2
(TAA)
(TASO)
TASO (mM)
D
TASO (mM)
TASO (mM)
E
PRC
Vinculin
- + - +
Oligomycin
Monocytes
DC
Live/Dead
SSC-A
CD14
FSC-A
FSC-W
CD1a
F
