# Supplementary material for: The regulatory role of PGC1α‐related coactivator in response to drug‐induced liver injury
Source: FASEB Bioadv. 2020 Jul 11;2(8):453–63. doi: 10.1096/fba.2020-00003 (PMC7429352; doi:10.1096/fba.2020-00003)
Supplement: Supplementary file 4 — Fig S4 [file FBA2-2-453-s004.pptx]

## Slide 1
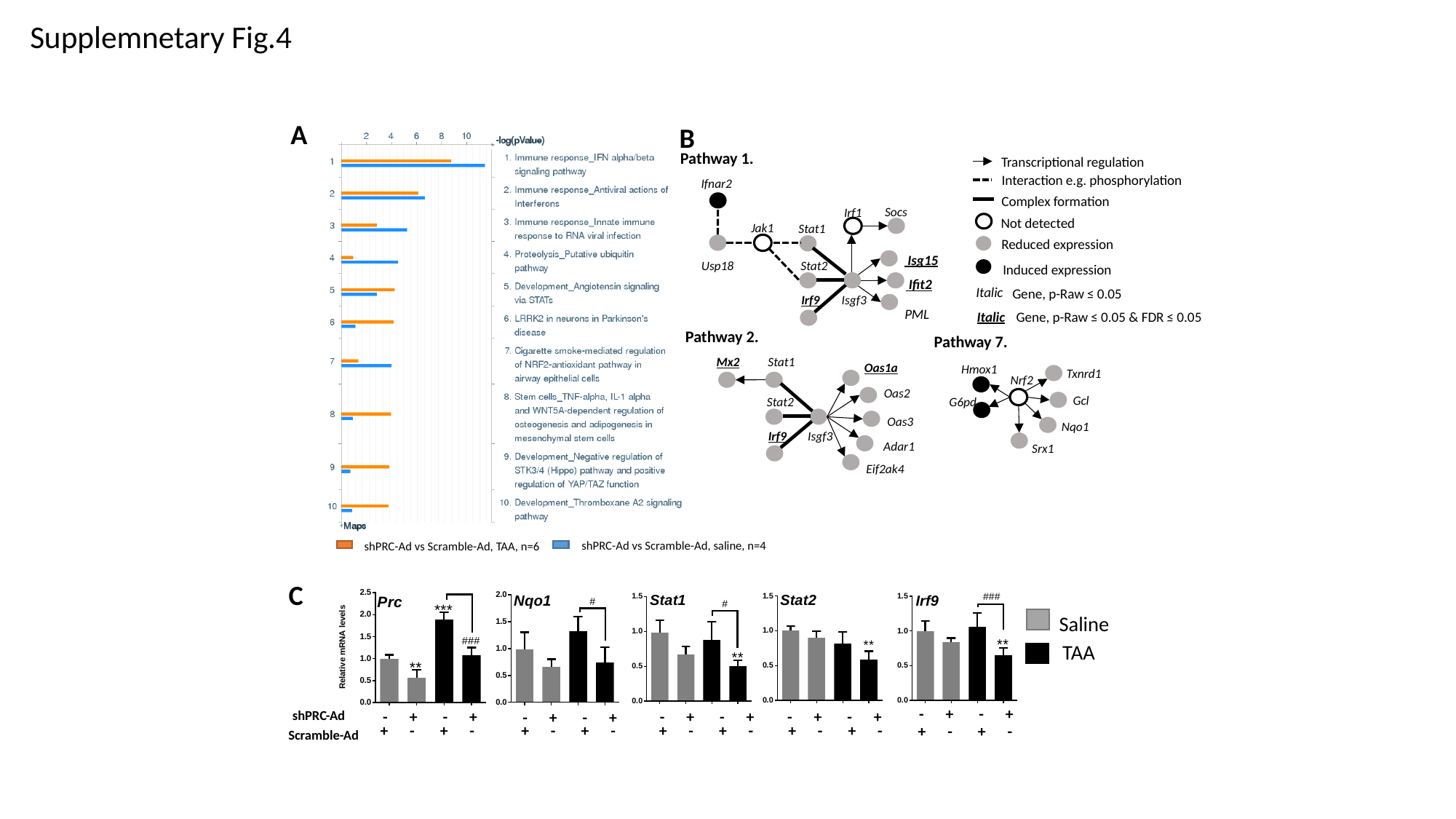

Supplemnetary Fig.4
A
B
Pathway 1.
Transcriptional regulation
Interaction e.g. phosphorylation
Ifnar2
Socs
Irf1
Jak1
Stat1
 Isg15
Usp18
Stat2
 Ifit2
Irf9
Isgf3
 PML
Complex formation
Not detected
Reduced expression
Induced expression
 Italic
Gene, p-Raw ≤ 0.05
Italic
Gene, p-Raw ≤ 0.05 & FDR ≤ 0.05
Pathway 2.
Pathway 7.
Mx2
Stat1
Oas1a
Oas2
Stat2
Oas3
Irf9
Isgf3
Adar1
Hmox1
Txnrd1
Nrf2
Gcl
G6pd
Nqo1
Srx1
Eif2ak4
shPRC-Ad vs Scramble-Ad, saline, n=4
shPRC-Ad vs Scramble-Ad, TAA, n=6
C
Saline
TAA
 - + - +
shPRC-Ad
 - + - +
 - + - +
 - + - +
 - + - +
 + - + -
 + - + -
 + - + -
 + - + -
 + - + -
Scramble-Ad
