# Supplementary material for: The regulatory role of PGC1α‐related coactivator in response to drug‐induced liver injury
Source: FASEB Bioadv. 2020 Jul 11;2(8):453–63. doi: 10.1096/fba.2020-00003 (PMC7429352; doi:10.1096/fba.2020-00003)
Supplement: Supplementary file 5 — Table S1 [file FBA2-2-453-s005.pdf]

Supp. Tab 1. List of TaqMan probes for qPCR.

**gene**	**species**	**TaqMan probe**
Actin β	*Mus musculus*	*Mm006077939_s1*
Prc	*Mus musculus*	*Mm00521078_m1*
NfκB1 (p100/p52)	*Mus musculus*	*Mm00476361_m1*
NfκB2 (p105/p50)	*Mus musculus*	*Mm00479807_m1*
Interleukin 1β	*Mus musculus*	*Mm00434228_m1*
Interleukin 6	*Mus musculus*	*Mm00446190_m1*
Interferon γ	*Mus musculus*	*Mm01168134_m1*
Tnfα	*Mus musculus*	*Mm00443258_m1*
Interleukin 10	*Mus musculus*	*Mm01288386_m1*
Ccl2	*Mus musculus*	*Mm00441242_m1*
Ccl5	*Mus musculus*	*Mm01302427_m1*
Cxcl1	*Mus musculus*	*Mm04207460_m1*
Cxcl10	*Mus musculus*	*Mm00492679_m1*
PRC	*Homo sapiens*	*Hs00209379_m1*
TNFα	*Homo sapiens*	*Hs_00174128_m1.*
