# Supplementary material for: The regulatory role of PGC1α‐related coactivator in response to drug‐induced liver injury
Source: FASEB Bioadv. 2020 Jul 11;2(8):453–63. doi: 10.1096/fba.2020-00003 (PMC7429352; doi:10.1096/fba.2020-00003)
Supplement: Supplementary file 7 — File S2 [file FBA2-2-453-s007.docx]

**Enrichment Analysis Workflow 1.0 Data Analysis Report**

*Server: portal.genego.com*

*Date: 2017‑01‑04*

# Experiments

| 1. |  | shPRC_TAA_stats_v2_TAA:VIRUS => TAA ‑> shPRC vs scramble.FoldChange |
| --- | --- | --- |
| 2. |  | shPRC_TAA_stats_v2_TAA:VIRUS => vehicle ‑> shPRC vs scramble.FoldChange |

The experiments uploaded for comparative analysis

# Table of content:

**[•](#Bookmark_1)** [Enrichment analysis](#Bookmark_1)

**[•](#Bookmark_2)** [Pathway Maps](#Bookmark_2)

**[•](#Bookmark_3)** [Top maps (sorted by Statistically significant Maps)](#Bookmark_3)

**[•](#Bookmark_4)** [1. Map : Immune response_IFN alpha/beta signaling pathway](#Bookmark_4)

**[•](#Bookmark_5)** [2. Map : Immune response_Antiviral actions of Interferons](#Bookmark_5)

**[•](#Bookmark_6)** [3. Map : Immune response_Innate immune response to RNA viral infection](#Bookmark_6)

**[•](#Bookmark_7)** [4. Map : Proteolysis_Putative ubiquitin pathway](#Bookmark_7)

**[•](#Bookmark_8)** [5. Map : Development_Angiotensin signaling via STATs](#Bookmark_8)

**[•](#Bookmark_9)** [Process Networks](#Bookmark_9)

**[•](#Bookmark_10)** [Diseases (by Biomarkers)](#Bookmark_10)

**[•](#Bookmark_11)** [GO Processes](#Bookmark_11)

## Enrichment analysis ([TOC](#TOC_table))

Enrichment analysis consists of matching gene IDs of possible targets for the "common", "similar" and "unique" sets with gene IDs in functional ontologies in MetaCore. The probability of a random intersection between a set of IDs the size of target list with ontology entities is estimated in p‑value of hypergeometric intersection. The lower p‑value means higher relevance of the entity to the dataset, which shows in higher rating for the entity.

Ontologies available for EA in Enrichment Analysis Workflow:

### Pathway Maps ([TOC](#TOC_table))

Canonical pathway maps represent a set of signaling and metabolic maps covering human in a comprehensive way. All maps are created by Thomson Reuters scientists by a high‑quality manual curation process based on published peer‑reviewed literature. Experimental data is visualized on the maps as blue (for downregulation) and red (upregulation) histograms. The height of the histogram corresponds to the relative expression value for a particular gene/protein.


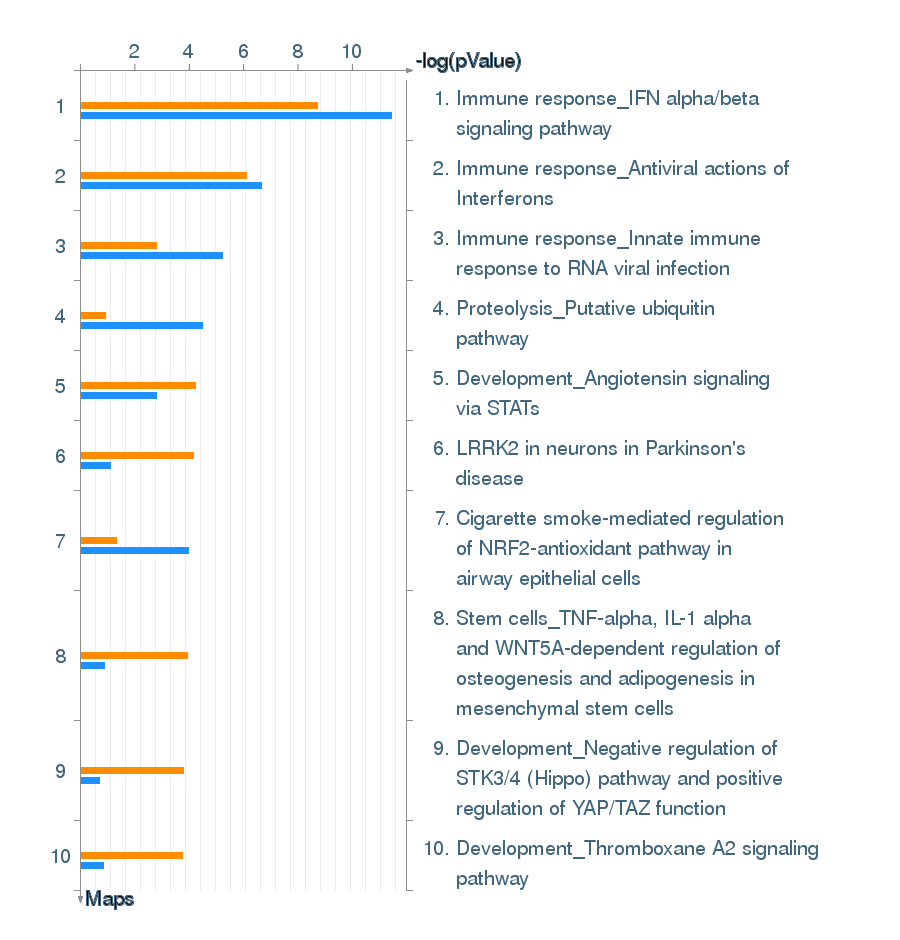


**Figure 2.** Pathway Maps. Sorting is done for the 'Statistically significant Maps'.

### Top maps (sorted by Statistically significant Maps)

**1. Map :** [Immune response_IFN alpha/beta signaling pathway](https://portal.genego.com/cgi/imagemap.cgi?id=429) ([TOC](#TOC_table))


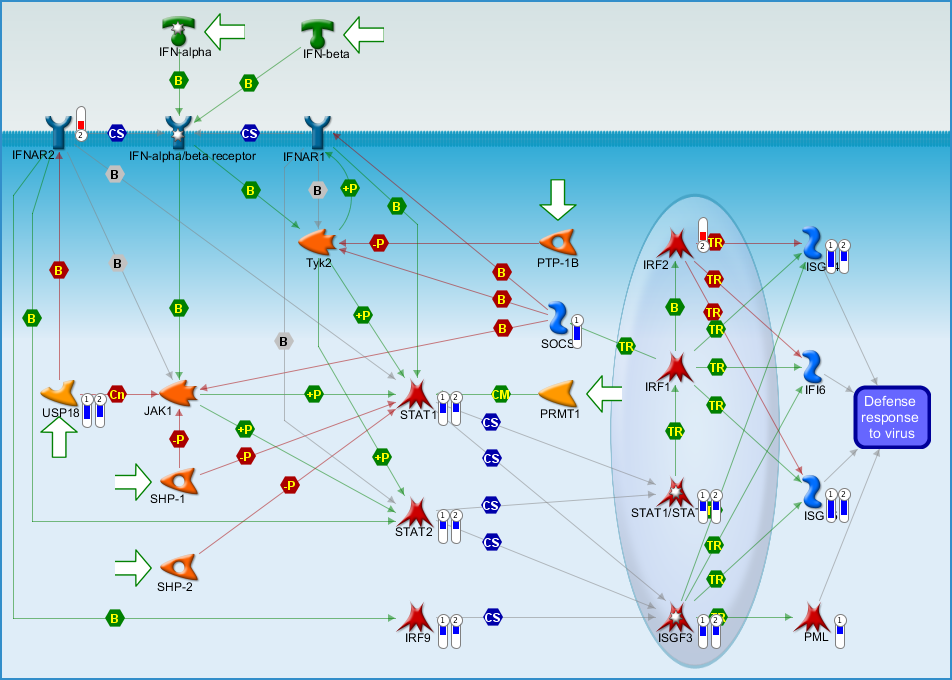


**Figure 3.** The top scored map (map with the the lowest p‑value) based on the enrichment distribution sorted by 'Statistically significant Maps' set. Experimental data from all files is linked to and visualized on the maps as thermometer‑like figures. Up‑ward thermometers have red color and indicate up‑regulated signals and down‑ward (blue) ones indicate down‑regulated expression levels of the genes.

**2. Map :** [Immune response_Antiviral actions of Interferons](https://portal.genego.com/cgi/imagemap.cgi?id=2225) ([TOC](#TOC_table))


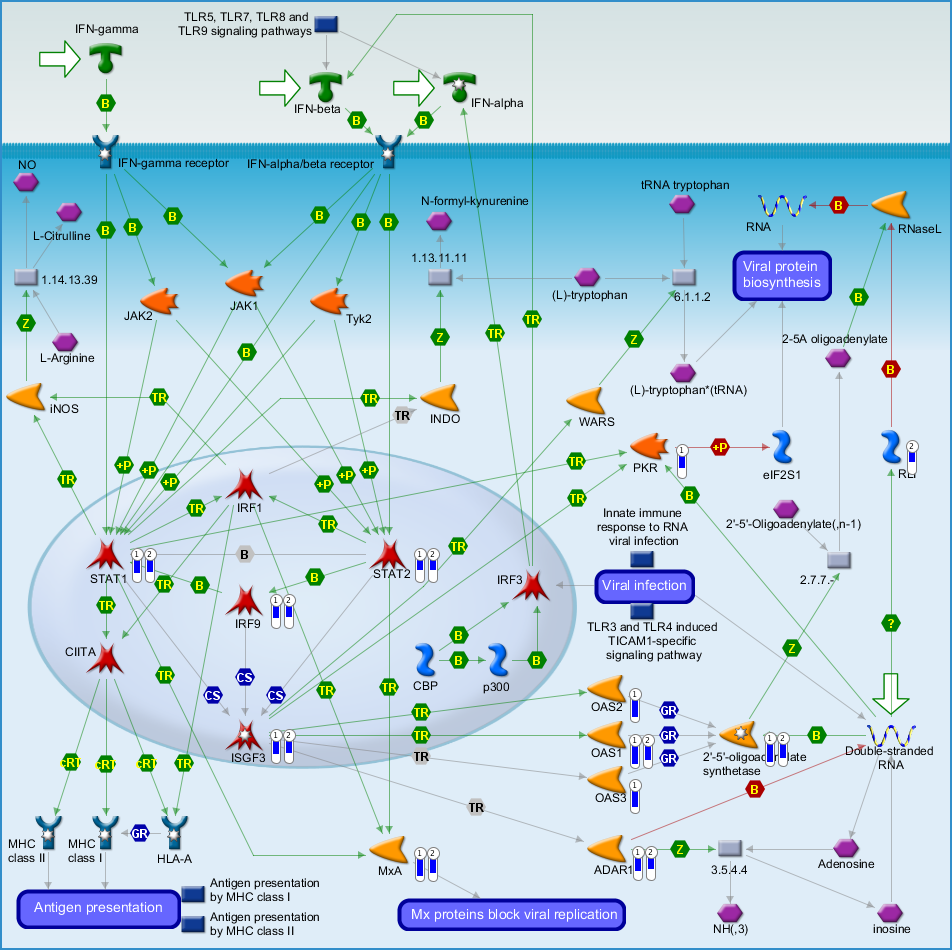


**Figure 4.** The second scored map (map with the second lowest p‑value) based on the enrichment distribution sorted by 'Statistically significant Maps' set. Experimental data from all files is linked to and visualized on the maps as thermometer‑like figures. Up‑ward thermometers have red color and indicate up‑regulated signals and down‑ward (blue) ones indicate down‑regulated expression levels of the genes.

**3. Map :** [Immune response_Innate immune response to RNA viral infection](https://portal.genego.com/cgi/imagemap.cgi?id=3076) ([TOC](#TOC_table))


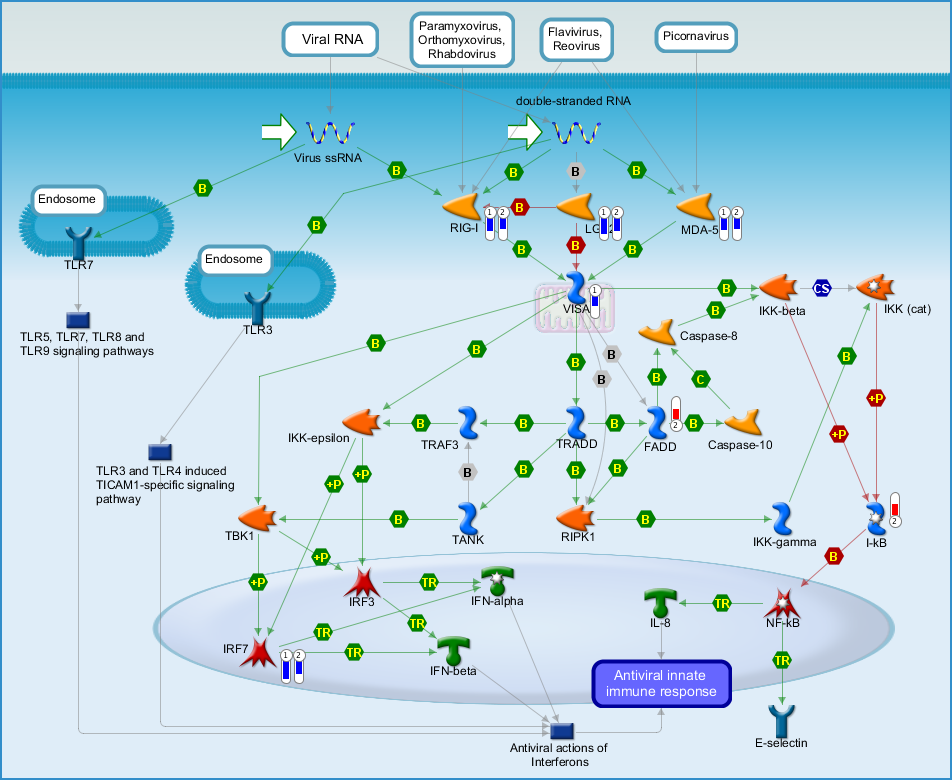


**Figure 5.** The third scored map (map with the third lowest p‑value) based on the enrichment distribution sorted by 'Statistically significant Maps' set. Experimental data from all files is linked to and visualized on the maps as thermometer‑like figures. Up‑ward thermometers have red color and indicate up‑regulated signals and down‑ward (blue) ones indicate down‑regulated expression levels of the genes.

**4. Map :** [Proteolysis_Putative ubiquitin pathway](https://portal.genego.com/cgi/imagemap.cgi?id=700) ([TOC](#TOC_table))


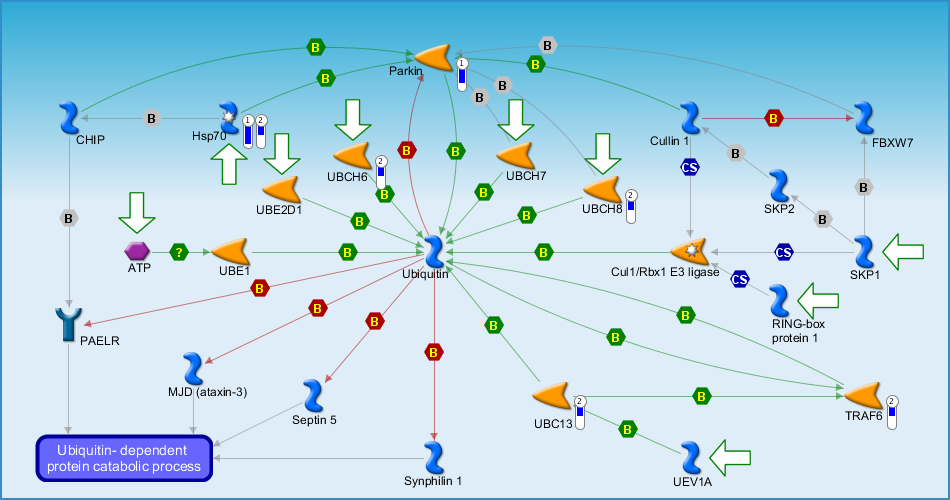


**Figure 6.** The fourth scored map (map with the fourth lowest p‑value) based on the enrichment distribution sorted by 'Statistically significant Maps' set. Experimental data from all files is linked to and visualized on the maps as thermometer‑like figures. Up‑ward thermometers have red color and indicate up‑regulated signals and down‑ward (blue) ones indicate down‑regulated expression levels of the genes.

**5. Map :** [Development_Angiotensin signaling via STATs](https://portal.genego.com/cgi/imagemap.cgi?id=439) ([TOC](#TOC_table))


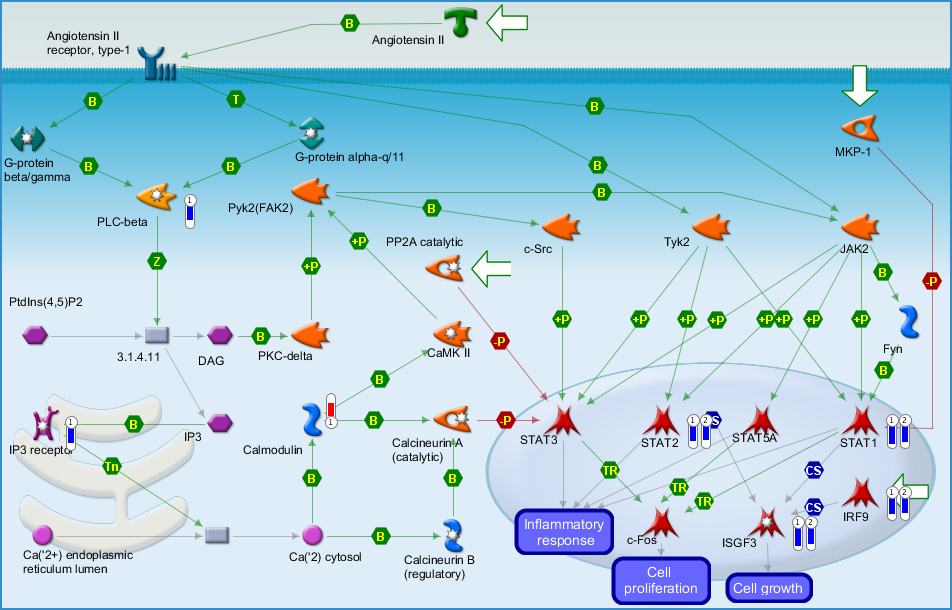


**Figure 7.** The fifth scored map (map with the fifth lowest p‑value) based on the enrichment distribution sorted by 'Statistically significant Maps' set. Experimental data from all files is linked to and visualized on the maps as thermometer‑like figures. Up‑ward thermometers have red color and indicate up‑regulated signals and down‑ward (blue) ones indicate down‑regulated expression levels of the genes.

### Process Networks ([TOC](#TOC_table))

The content of these cellular and molecular processes is defined and annotated by Thomson Reuters scientists. Each process represents a pre‑set network of protein interactions characteristic for the process.


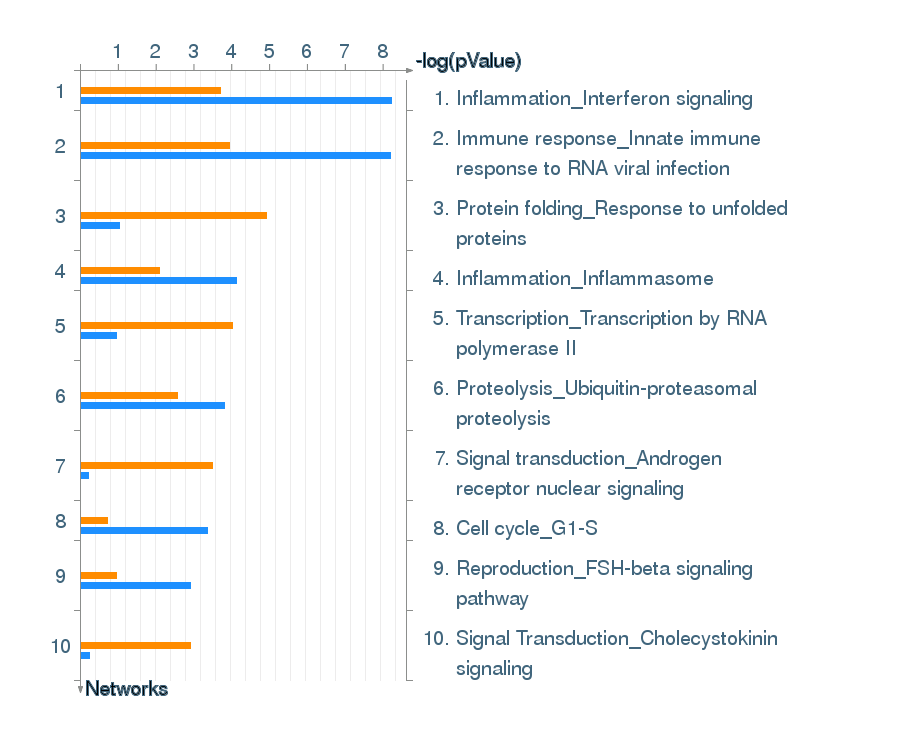


**Figure 8.** Process Networks. Sorting is done for the 'Statistically significant Networks'.

### Diseases (by Biomarkers) ([TOC](#TOC_table))

Disease folders are organized into a hierarchical tree. Gene content may very greatly between such complex diseases as cancers and some Mendelian diseases. Also, coverage of different diseases in literature is skewed. These two factors may affect p‑value prioritization for diseases.


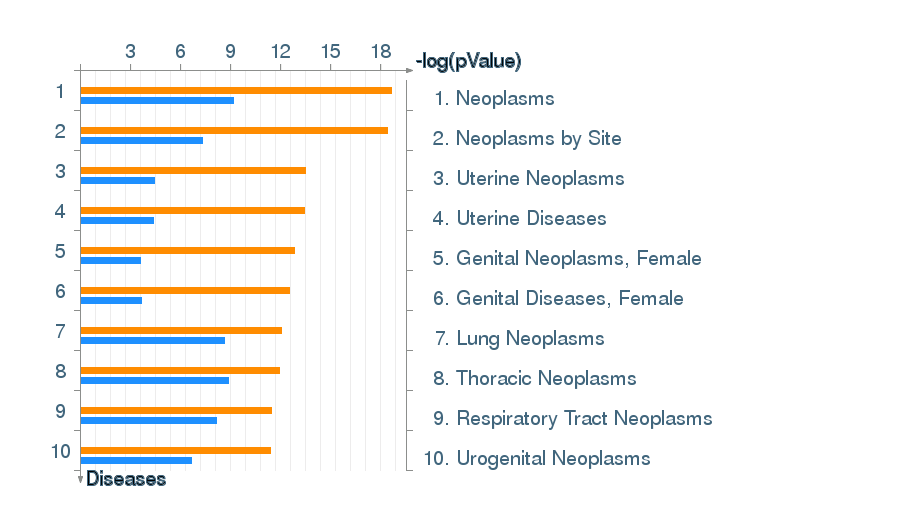


**Figure 9.** Diseases (by Biomarkers). Sorting is done for the 'Statistically significant Diseases'.

### GO Processes ([TOC](#TOC_table))

These are Gene Ontology (GO) cellular processes. As most GO processes have no gene/protein content, the "empty terms" are excluded from p‑value calculations.


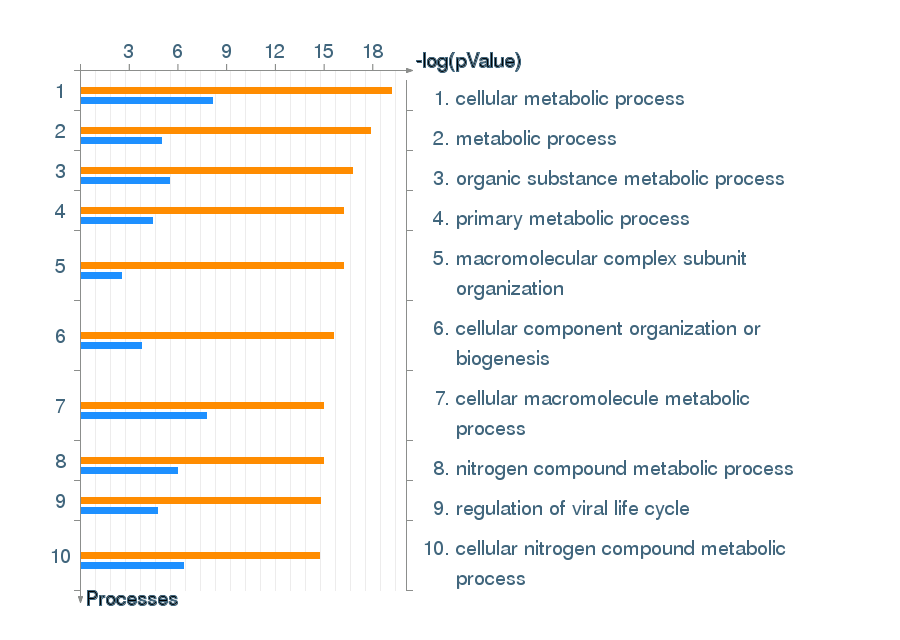


**Figure 10.** GO Processes. Sorting is done for the 'Statistically significant Processes'.
