# Supplementary material for: The regulatory role of PGC1α‐related coactivator in response to drug‐induced liver injury
Source: FASEB Bioadv. 2020 Jul 11;2(8):453–63. doi: 10.1096/fba.2020-00003 (PMC7429352; doi:10.1096/fba.2020-00003)
Supplement: Supplementary file 10 — Supplementary Material [file FBA2-2-453-s010.pdf]

Supplementary figures legends

Supplementary Fig. 1. Effect of TAA treatment in mouse liver.

A) Female mice were treated with either TAA or vehicle by i.p injection as indicated, and tissue and plasma samples were collected 24h later. Total liver protein was separated on SDS-PAGE gel and hepatic Cyp2E1 (A) and PRC (B) levels were evaluated with appropriate antibodies using Western blotting. Anti-Vinculin ab were used as a loading control. Vinculin normalized PRC and Cyp2e1 levels are shown. C) mRNA expression of *Prc* and selected hepatic immune system factors in the same animals D) Circulating levels of ALT and AST E) Relative number of monocytes and neutrophils in the liver presented as percentage of CD45+ cells. Statistically significant changes compared to control (saline) animals was analyzed using ANOVA, followed by Dunnett's test (n=4) * * $p<0.05$, ** $p<0.01$, *** $p<0.001$, SD. Effect of different doses of shPRC-Ad in the liver. F) Male mice were injected with saline or two different doses of scramble-Ad or shPRC-Ad adenoviruses (VP/kg). Animals were sacrificed 3 days later and expression of selected genes measured by qPCR, n=2 G) ALT and AST levels from the same animals

Supplementary Fig. 2.

A) Representative flow cytometry plots of the liver.

Live, CD45+ cells were gated as follows: B cells (TCRβ^{-}CD19^{+}), CD4 T cells (CD19^{-}TCRβ^{+}CD4^{+}), CD8 T cells (CD19^{-}TCRβ^{+}CD8^{+}), neutrophils (CD19^{-}TCRβ^{-}Ly6g^{+}Ly6c^{int}), eosinophils (CD19^{-}TCRβ^{-}F4/80^{+}SSC^{hi}), monocytes (CD19^{-} TCRβ^{-}Ly6c^{+}Ly6g^{-}CD11b^{+}) and dendritic cells (CD19^{-} TCRβ^{-}CD11c^{hi} MHCII^{hi}). Data is representative of 3 experiments.

B) Expression of selected activation markers on hepatic monocytes in mice infected with shPRC-Ad.

Male mice were infected with scramble or shPRC adenovirus ($3*10^{12}$ VP/kg) for 48h and treated with 100 mg/kg TAA for 24h before sacrifice. Single cell suspensions were isolated from the liver of these mice as described and submitted to FACS analysis. Monocytes were gated as CD45^{+} Ly6G^{-} F4/80^{mid}, CD11b^{+} and Ly6C^{hi} (illustrated in Sup. Fig. 3) and the expression of CD80, CD86, CD40 and PDL1 on Ly6C^{+} liver monocytes was determined using specific antibodies (anti-CD80 PE antibody, clone: 16-10A1, BD; anti-CD86 APC-Cy7 antibody, clone: GL1, Biolegend; anti-CD40 BV786 antibody, clone: 3/23, BD; anti-PDL-1

BV421 antibody, clone: 10F9G2, Biolegend). Expression was determined and plotted as mean fluorescence intensity (MFI). Two tailed t-test, *P<0.05, **P<0.01, ***P<0.001, n=4-6. Representative of two independent experiments is shown.

Supplementary Fig. 3.

A-E) Effect of TAA, TASO and oligomycin on PRC expression in HepG2 cells.

HepG2 cells were treated with TAA (A) or TASO (B) as indicated for 24h and PRC protein expression measured using Western blotting. Vinculin was used as a loading control and Vinculin normalized PRC levels were quantified. ANOVA, Dunnett's test, A) n=4, B) n=3 * denotes statistical significance to control * P<0.05, ** P<0.01. C) Schematic representation of CYP2E1 metabolizing TAA to TASO. D) Relative *PRC* and *TNFα* mRNA levels were quantified using qPCR in HepG2 cells treated with TASO as indicated for 6h. ANOVA Dunnett's test, n=3 * P<0.05, *** P<0.001, SD E) PRC levels in HepG2 cells treated with 0.04μM oligomycin for 24h. One tail t-test, *** P<0.001, SD. Representative of at least two independent experiments are shown.

F) Representative flow cytometry plots of the monocytes and dendritic cells.

Human blood PBMCs were isolated and cultured in the presence of GM-CSF and IL-4 to generate DCs as described in the materials and methods section. After 7 days, cells were harvested and monocytes and DCs were identified via flow cytometry. Within the gate of living cells, monocytes and DC were identified as CD14+CD1a- and CD14+CD1a+ cells respectively.

G, H) Effect of PRC knockdown in CCCP treated HepG2 cells on CD86 expression in human DC and monocytes.

HepG2 cells were transfected with either esiGFP or esiPRC for 24h prior 24h long treatment with 30μM CCCP and total homogenates collected (G). Vinculin was used as a loading control and PRC levels quantified. H) Human monocyte derived Dendritic Cells and monocytes were cultured for 24h in supernatants from HepG2 treated as in panel A, Mean Fluorescence Intensity (MFI) is shown. Panel A and B * denotes statistical significance to esiGFP control samples. # represents statistical significance between esiGFP and esiPRC samples treated with CCCP. Tukey's multiple comparisons test, G) n=3, H) n=7-8.

I, J) Effect of PRC knockdown in HepG2 cells treated with Oligomycin or CCCP on CD83 expression on human DC and monocytes. Human monocyte derived Dendritic Cells and monocytes were cultured for 24h in supernatants from HepG2 cells transfected with esiGFP or esiPRC1 for 24h and treated for additional 24h either with 0.04µM Oligomycin (I) or 30µM CCCP (J). Mean Fluorescence Intensity (MFI) is shown. * denotes statistical significance to esiGFP control samples. # represents statistical significance between esiGFP and esiPRC samples treated with compounds ANOVA, Tukey's multiple comparisons test, n=7-8.

Supplementary Fig. 4.

Hepatic pathways modulated by Prc A) Pathway enrichment analyses were done using the MetaCore analysis software and the top significantly enriched pathways in mice infected with scramble-Ad or shPRC-Ad adenoviruses and treated with TAA (orange) or saline (blue) for 24h are listed. B) Schematic representation of pathways number 1, 2 and 7 from panel A. C) qPCR results of selected genes from the same samples. ANOVA, Tukey´s test, n=4-6, * denotes statistical significance to saline injected scramble-Ad group while # relates to scramble-Ad or shPRC-Ad treated with TAA, * $P<0.05$, ** $P<0.01$, *** $P<0.001$, #$P<0.05$, ## $P<0.01$, ###$P<0.001$, SD

Supplementary files

Supp. File 1 FDR, fold change and p-Raw values of *Prc* mRNA expression data obtained from TG-Gate portal

Supp. File 2 Characterization of 5 top scoring hepatic pathways from Metacore

Supp. File 3 Enrichment analysis report of top scoring hepatic pathways from Metacore Pathway

Supp. File 4 List of genes used for Metacore pathway enrichment analysis, containing FDR and p-Raw values
